# Sustainable Supply Chain Strategy and Sustainable Competitive Advantage: A Mediated and Moderated Model

**DOI:** 10.3389/fpubh.2022.895482

**Published:** 2022-05-19

**Authors:** Jianmin Sun, Muddassar Sarfraz, Kausar Fiaz Khawaja, Muhammad Ibrahim Abdullah

**Affiliations:** ^1^School of Management, Nanjing University of Posts and Telecommunications, Nanjing, China; ^2^School of Management, Zhejiang Shuren University, Hangzhou, China; ^3^Faculty of Management Sciences, International Islamic University, Islamabad, Pakistan; ^4^Department of Management Sciences, COMSATS University Islamabad, Lahore, Pakistan

**Keywords:** sustainable supply chain, strategy, sustainability inhibitors, sustainable supply chain practice, sustainable competitive advantage

## Abstract

In the global business environment, sustainability has become a competitive priority of most enterprises. Sustainability as a differentiation approach enables organizations to compete in today's environment. However, despite the increasing adaptability to sustainability practices, pharmaceutical supply chain management is still facing challenges in preserving the global environment. The study contemplates the impact of sustainable supply chain strategy and sustainable competitive advantage while considering the mediating role of sustainable supply chain practice and moderating role of sustainability inhibitors. The data was collected from the 180 employees working in the pharmaceutical companies of Pakistan. The study applied a quantitative approach for testing a theoretical model, using discriminant validity analysis, assessment of measurement model, composite reliability & validity analysis to assess the dimensions and model's reliability and validity. The study results show a positive relationship between sustainable supply chain strategy and sustainable competitive advantage. Sustainable supply chain practice mediates the relationship between sustainable supply chain strategy and sustainable competitive advantage, while sustainability inhibitors strengthen the relationship between sustainable supply chain strategy and sustainable supply chain practice. This study will help managers develop impactful sustainable supply chain management practices that will lead to sustainable performance.

## Introduction

Significantly, in recent years, the issue of sustainable development has become a fast-growing phenomenon, gaining the interest of global businesses and communities. As a result, today, the accelerating market competition and progressing globalization have drastically altered the supply chain demand, leading the government and stakeholders to encourage the integration of sustainable practices. Substantially, this developing concept led organizations to design business strategies (i.e., social, economic, and ecological), thereby focusing on supply chain sustainability (1).

Indeed, today's market instability has profoundly called organizations for sustainable development. This current scenario has encouraged most companies to embrace sustainable supply chain strategies (SSCS) for achieving their business targets. In explaining this notion, the research shows that, in supply chain management, designing a sustainable business strategy helps the organization achieve its business objectives (2). Accordingly, a sustainable supply chain framework indicates that firms' strategies help them achieve sustainable goals and management support (3). Perhaps, understanding the significance of growing sustainability inspires the management to focus on developing sustainable strategy designs (4). Hence, this sustainability idea demands organizations to integrate sustainable supply chain strategies across the organizational levels. SSC makes the organizations serve different sustainability dimensions (i.e., social, economic, environmental), thereby making the firm hold an invincible market position (5). Therefore, in studying the impact of sustainable strategy, it has become imperative to recognize its link with a sustainable competitive advantage.

Additionally, in the strategic alignment to SSCS, the sustainability concept allows the firms to achieve a distinct advantage, matching the firms' business goals. The sustainable competitive advantage (SCA) fosters the companies' performance, thereby making organizations outperform their peers. Given the articulation, the study shows that the enduring competitive advantage improves firms' performance, financial outcomes, and employees' commitment (6). Furthermore, the company's strategic tool (i.e., SCA) also encourages achieving the triple sustainability dimension (i.e., economic, social, ecological). Given the statement, the study shows that companies embracing supply chain strategies achieve a superior advantage in the hyper-dynamic business environment (7). Altogether, this concept of balanced sustainability increasing adds to the firms' value, thus ensuring long-term advancement and competitiveness (8).

However, besides the growing sustainability advantage, the medical industry is still encountering challenges regarding supply chain stability. The pharmaceutical division is the most lightened industrial sector that demands sustainability in serving humanity. The pharmaceutical bestowed the responsibility to deliver life-saving products to humanity. Given the articulation, the study shows that the industry's production (e.g., material, products, services) extensively influences societies' wellbeing (9). In particular, the medicine industry holds a prominent position among the global economies, highlighting the need for enduring strategies. In serving society, the literature shows that pharmaceutical companies are increasingly facing problems such as medical waste, thus requiring the need for sustainable strategies for securing the global environment and societies' (10). Perhaps, based on this statement, the prior study shows that the pharmaceutical sector concerning the supply chain remains underdeveloped (11).

In particular, sustainability, the foremost agenda, elevates the need for firms' sustainable practices. SSCS plays a critical role in promoting sustainable practices. It serves as the ground for developing SSCP, thus achieving organization goals (12). Moreover, SSCPs make the organization achieve a multi-dimensional sustainability purpose. Given the explanation, the study states that sustainable practices ensure firms' enduring performance (e.g., eco-friendly practices, green products) (13). Similarly, the study also states that these positive sustainability dimensions improve the firms' practices (14), thereby achieving an enduring advantage.

Moreover, in ensuring enduring practices, numerous factors associated with firms' stability require consideration. However, without an adequate understanding of eco-friendly inhibitors, it is difficult for organizations to evaluate their sustainability practices. Sustainability inhibitors are naturally occurring substances that limit the corrosion of intoxicant materials. The intoxicant inhibitors are a barrier to achieving firms' sustainability. The usage of eco-friendly inhibitors ensures an improvement in the environment (15). These latest developments save humanity and the environment from harm. Given the illustration, the study shows that different eco-friendly alternatives (e.g., medicines, drugs, toxic-free chemicals) are extensively adopted to enhance the firms' sustainability practices (16). Hence, organic inhibitors are an essential alternative to improving firms' practices (17). Indeed, the literature depicts these sustainable inhibitors as the prime development of today's era.

However, in recent years, the initiative of sustainable development has massively captured the attention of most pioneer organizations. However, the literature indicates that it has become difficult for the pharmaceutical industry to sustain itself in today's competitive environment. In particular, the literature concludes that despite the increasing growth of SSC, its importance in the pharmaceutical industry still needs attention. Moreover, the above considerations demand investigation in the medical sector compared to other divisions. The literature states that sustainable supply chain practices are of high complexity in the supply chain (18), which colossally needs investigation.

Therefore, against this drawback, this study contributes to SSCM by determining the impact of organizational sustainability from a competitive advantage perspective. Significantly, this study fulfills the research gap by investigating the relationship between the sustainable supply chain strategy and sustainable competitive advantage. Similarly, it also analyzes the mediating role of sustainable supply chain practices and moderating role of sustainability inhibitors nexus the relationship between SSCS and SCA.

Fundamentally, this study is multidisciplinary research that encourages implementing the sustainability concept in gaining a competitive advantage. In particular, this paper extensively shifts the focus of several scholars to sustainable supply chain management. It is a significant study that incorporates the concepts of supply chain and sustainability, thereby elevating the need for achieving sustainable advantage. Moreover, it includes the dimensions of sustainability under the supply chain context, thereby making organizations understand the sustainability approaches to improving the competitive advantage. Indeed, this current understanding of SSC is of colossal importance to organizations. It encourages firms to align their business practices to the strategic plan of achieving long-term functionality. Moreover, this study seeks the best approaches to enhancing the firms' sustainability by suggesting the governments, stakeholders, management, and organizations comply with environmentally friendly strategies to gain distinctive advantages.

The first section introduces the research topic by defining the research objectives and significance. Furthermore, the literature review (i.e., Section Hypothesis Development) presents the background of the study. In the same vein, Section Study Methodology describes the research tools and methods. After, the subsequent section documents the study findings, with Section Discussion discussing the outcomes in the light of empirical literature. Finally, the paper ends with the conclusion, research limitations, and future directions.

## Hypothesis Development

### Sustainable Supply Chain Strategy and Sustainable Competitive Advantage

Over the years, the increasing globalization has massively emphasized the adoption of sustainable supply chain strategies, fundamentally opening new avenues in the era of increased competitiveness. However, understanding this source of sustainable competitiveness has led organizations to embrace novel strategies for accelerating firms' advancement. The sustainable supply chain strategy is a robust strategical plan that helps firms defeat the market rivalry, thus gaining a sustainable competitive edge. Given the illustration, the study states that the sustainability approach develops value across the production chain, potentially discovering new sources for distinctive advantages (19). Accordingly, the study appreciating SSCS as a sustainable benefit states that in the supply chain network, the sustainable strategies enable the organizations to foster competitiveness, thus acquiring sustainable competitive advantage (20).

In particular, the effective SSCS enables the firms to harmonize their business activities, thereby strategically intensifying their business position. Given the illustration, the research reveals that the sustainability triple-bottom-line dimensions (e.g., social, economic, and ecological) make SSCS accomplish a dominant position in the marketplace (5). As a result, today, sustainability strategies have become critical to function internally, thereby accomplishing the organization's sustainable goal (21). The key to gaining sustainability is to sustain in the increasingly competitive environment, substantially obtaining long-term competitive stability. In the supply chain business environment, the SSCS facilitates a business operation, proactively regulating market changes. Based on this statement, the study shows that the SSCS provides competitive benefits to the firms', thus making them operate in a dynamic business environment (22). Significantly, the SSCS provides a first-mover advantage to the firms by getting ahead of other firms (23). Hence, based on this literature, we conclude the following hypothesis:

***H1****: Sustainable supply chain strategy has a positive and significant impact on sustainable competitive advantage*.

### Sustainable Supply Chain Strategy and Sustainable Supply Chain Practice

In recent years, the increasing environmental concerns have made the sustainability practices receive management attention. In the supply chain, ecological issues severely affect the value chain system, thus demanding the need for effective strategies and sustainability practices across the supply chain network. Therefore, today, numerous firms have adopted SSCS for improving ecological welfare. Given the articulation, the study shows that the firms' sustainability strategies reduce the environmental cost, thereby maximizing the social welfare with sustainable practices (24).

In particular, sustainable supply chain strategies assist the firms in attaining the sustainable triple-bottom-line dimension. In supply chain modeling, organizations' enduring plans and sustainable practices devote to improving the organizations' function. Perhaps, understanding this multi-dimensional sustainability helps companies to support organizations' practices with a sustainability vision. The firms' sustainability strategies lead to the effective implementation of sustainable practices. For this reason, many companies have started embracing the concept of sustainable practices complementing the firms' sustainable goals (3). In the illustration, the study shows that the SSCP makes the organizations achieve the sustainability objectives by exhibiting superior organizational performances (25).

Arguably, many issues elevate the demand for sustainable approaches. In the pharmaceutical industry, to reach sustainability standards, companies have realized the need for sustainability practices to accomplish a business goal. The effort to improve the competitiveness in the pharmaceutical industry has made the organizations implement sustainability practices, significantly minimizing the increasing impact of ecological concerns (i.e., chemical waste, inhibitors) (25). In particular, according to the world health organization, the support of environmentally friendly strategic plans, strategies, practices, and policies help the organization guide the management, thus securing the environment (26). Consequently, based on the prior consideration, we have developed the following hypothesis:

***H2****: Sustainable supply chain strategy has a positive and significant impact on sustainable supply chain practice*.

### Sustainable Supply Chain Practice and Sustainable Competitive Advantage

Over the past decades, environmental sustainability has received increasing significance in supply chain management. In recent years, the high escalation of environmental deterioration has made the stakeholders adopt eco-friendly practices for securing the long-term advantage. The firms' sustainable characteristics (e.g., actions, policies, processes) help firms to obtain a sustainable competitive advantage. Given the articulation, the study shows that the adoption of SSCP makes the companies encompass the sustainability dimensions, thus exhibiting high competitiveness and superior organizational performance (27).

Undoubtedly, competition is the most influential driver for incorporating sustainability in the supply chain. Organizations under its influence feel the pressure of adopting sustainable practices for gaining a competitive edge. The sustainable competitive advantage makes firms differentiate themselves from their rivals. Indeed, SSCP plays a vital role in maintaining market competitiveness. The sustainability practices enable the organizations to outperform their competitors by focusing on long-term stability. Given the illustration, the study found that sustainable supply chain practices provide the company with a sustainable competitive advantage with improved organizational performance (28).

However, the business's success largely depends on the firms' competitive benefit. By embedding the sustainability practices into firms' functions, the organizations can maintain a differential edge over its peer. Accordingly, the research shows that the SSCP enhances the firms' strategic assets, thus leading to SCA (29). Indeed, the supply chain sustainability practice leverages the firms' operations by providing competitive benefits. Therefore, due to its increasing significance (i.e., SSCP), the study suggests that companies should focus on sustainability practices to maintain a differential advantage over the other firms' (30). Therefore, based on these systematic reviews, our hypothesis states:

***H3****: Sustainable supply chain practice has a positive and significant impact on sustainable competitive advantage*.

### The Mediating Role of Sustainable Supply Chain Practices

Today, numerous organizations are increasingly facing challenges regarding environmental deterioration. In such a situation, the sustainability practices potentially help the companies to neutralize the prevailing damage. Significantly, the collaborative supply chain network exhibiting sustainability development had reduced the ecological footprint spoiling the motherland. Indeed, building on this concept, organizations' have ensured establishing supply chain sustainability on multiple levels for gaining sustainable competitive advantage (31). Given the illustration, the study showed that the supply chain had highlighted the demand for incorporating the sustainable agenda in the business strategy, thereby exploring the market benefits, performance indicators, and sustainable advantage (32).

However, investigating this agenda in the pharmaceutical company shows that SSCS makes the firms acquire long-term stability and competitive advantage (33) with cleaner practices. This strategy of boosting the firm's sustainability performance helps the organization recognize the significance of developing sustainable supply chain practices. However, developing sustainable supply chain strategies ensures the development of sustainable practices, capabilities, and processes driving the competitive advantage. Given the illustration, the study shows that SSCS provides an enduring edge to organizations by ensuring cleaner production practices (34). The implementation of sustainable practices varies from organization to organization. Sustainable practices improve the firms' vision, thus achieving higher competitive standards (35). In particular, the sustainability practices enable the firms to take over the market competition by providing a sustainable competitive edge. Hence, from the prior study findings, we have inferred the following hypothesis:

***H3a:*** *Sustainable supply chain practice mediates the relationship between sustainable supply chain strategy and sustainable competitive advantage*.

### The Moderating Role of Sustainability Inhibitors

The international supply chain has increasingly faced environmental issues that need human visibility. In particular, these complexities have facilitated the need for environmentally friendly approaches to balance the ecological footprint (36). Ecological sustainability demands adopting sustainable practices as the motivators for combating the barriers (i.e., unsustainable inhibitors). Unsurprisingly, the pharmaceutical industry is colossally affected by the adversity of intoxicate inhibitors, which forms the foundation of poor economic stability. Given the explanation, the study reveals that for combating the unsustainable inhibitors, eco-friendly tools (e.g., technology) had widely adopted, reducing the ecological waste, potentially fulfilling is the fundamental goal of firms' strategy (37).

Indeed, today organizations have widely emphasized the implementation of sustainable inhibitors as a part of the firms' strategy. Given the articulation, the study findings suggest that management should invest in sustainable supply chain practices (38) to minimize the adversity of unsustainable pollutants. In contrast, the study shows that the sustainable supply chain practice provides a theoretical lens to the firms' enabling them to relish the benefit of sustainability inhibitors (39). Accordingly, in the supply chain, the pharmaceutical industry produces considerable waste throughout the production process. This excessive ecological burden (e.g., drugs and intoxicants) makes the managers implement sustainability inhibitors throughout the manufacturing process. The pharmaceutical model of sustainability practices reduces the effect of non-value additions activities, thus eliminating the waste. Given the illustration, the study states that the sustainability inhibitors drive the sustainability practices (40) to improve the socio-ecological welfare. Subsequently, the hypothesis built on the above consideration concludes:

***H4****: Sustainability inhibitors strengthen or moderate the relationship between sustainable supply chain strategy and sustainable supply chain practice*.

**Figure 1:**
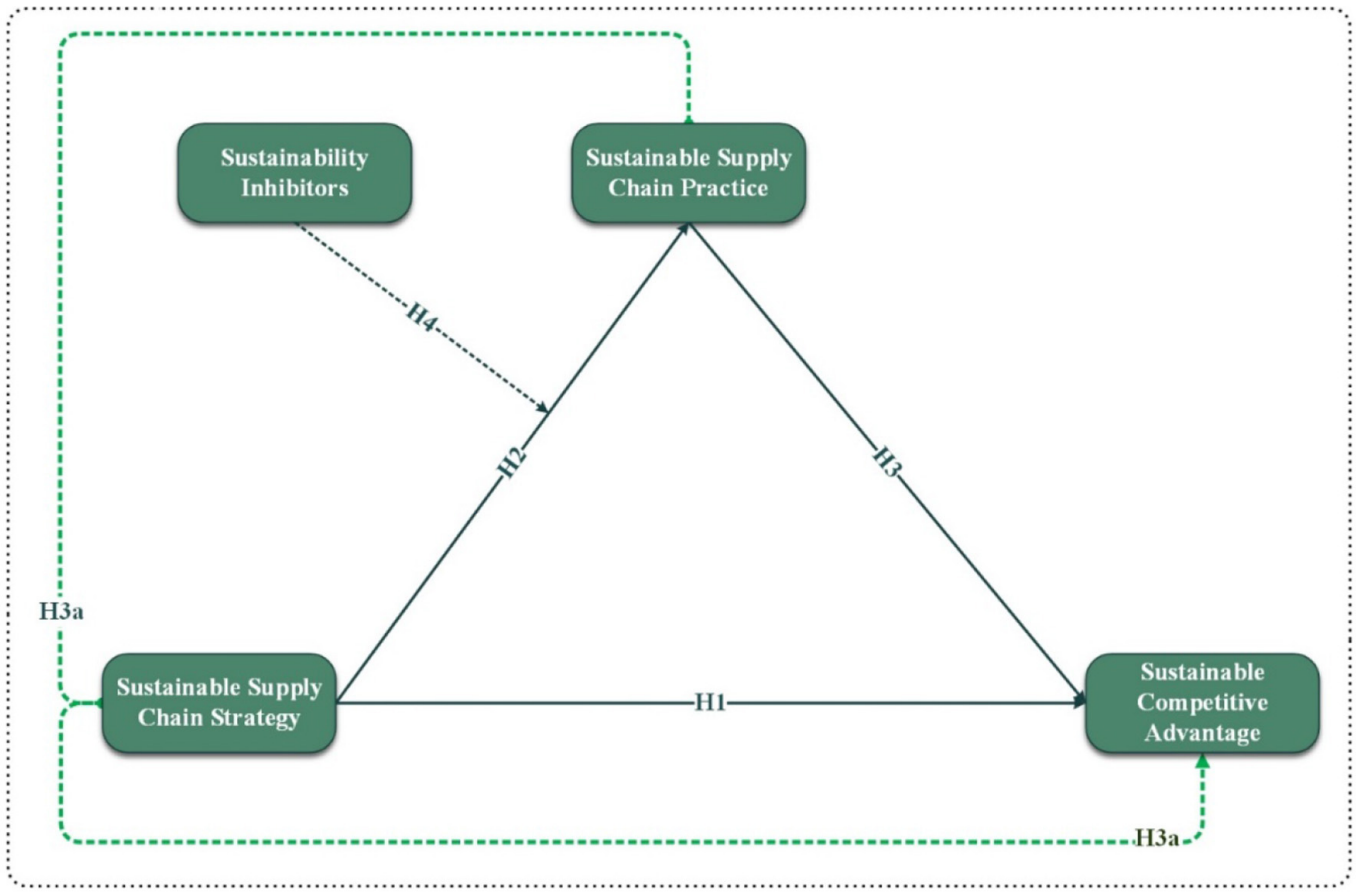
Conceptual framework.

Figure 1 shows the study's theoretical framework (independent, dependent, mediator, and moderator variables).

## Study Methodology

### Sample and Data Collection

The research adopted a survey research design to collect data to investigate the influence of sustainable supply chain strategy on sustainable competitive advantage, mediating the role of sustainable supply chain practice and moderating the role of Sustainability Inhibitors. Through the convenience sample technique, questionnaires were distributed among the managers of 25 pharmaceutical companies located in Pakistan; data was collected from January-February 2022. Approximately 250 managers were contacted and requested to participate in this time-lagged study, whereas 180 complete responses were received.

To control the common method bias, this research conducted a time-lagged study with 15 days intervals. Responses on sustainable supply chain strategy and sustainability inhibitors were collected at time 1; after a gap of 15 days, responses on sustainable supply chain practice were collected, i.e., time-2; and after a gap of 15 days, data was collected on sustainable competitive advantage, i.e., time-3. This research also applied the common method bias using Harman's single-factor approach. The variance extracted using one factor is 6.835%, less than 50%, indicating no common method bias in this study (41). Table 1 depicts the demographic characteristics of the study participants, revealing 52% male and 48% female. In total 13% of participants were in the age group 19–30; 28% were from 31–40. In total 36% of participants were Bachelor's degree holders, and 36% were master's.

**Table 1:** Demographic characteristics.

**Items**	**Frequency (*N* = 180)**	**(%)**
**Gender**		
Male	94	52.2
Female	86	47.8
**Age**		
19–30	24	13.3
31–40	48	26.7
41–50	50	27.8
51–60	32	17.8
>60	26	14.4
**Education**	
Intermediate	35	19.4
Bachelor	64	35.6
Master	64	35.6
MPhil/Others	17	9.4
**Marital status**	
Single	34	18.9
Married	146	81.1

### Measures

Sustainable supply chain strategy was measured using 3 item scale developed by Nayal et al. (42). Sample items include: “The industry markets the same products with minor variations as per consumer demand and trends of the market”; “The industry has relevant IT-related knowledge and skills to adapt to recent sustainable market trends.” Sustainability inhibitors was measured using 5 item scale developed by Gopal and Thakkar (43). Sample items include: “Resistance to technology advancement adoption” and “Unawareness of customers.” The dependent variable sustainable competitive advantage was measured using Nayal et al. (42) developed using a five-item scale. Sample items include: “Application of digital technologies and collaboration improves process efficiency”; “The digitalized Supply Chain responsible for improved collaboration and coordination improves flexibility.” Five-point Likert scale ranging from strongly disagree to strongly agree was used to measure the study variables. The mediating variable of sustainable supply chain practice was measured using a five-item scale developed by Gopal and Thakkar (43). It was measured using five Likert scales ranging from 1-not at all implemented 5-fully implemented. Sample items include: “Lean practices” and “Eco-design practices.”

### Assessment of Model Fit and Measurement Model

Data cleaning and screening were performed before conducting the reliability and validity analysis. Table 2 depicts the results of model fit, reliability, and validity analysis. Results of CFA- four-factor model depicts values within the acceptable range CFI = 0.999; TLI = 0.998; SRMR = 0.0394; NFI = 0.938; RMSEA = 0.011; hence confirming model fitness.

**Table 2:** Model fit and reliability and validity analysis.

**Model fit Indexes**					
**Fit Index**	**Cited**	**Fit criteria**	**Results**	**Fit (Yes/No)**	
X2			131.771		
DF			129		
X2/DF	(44)	1.00–5.00	1.021	Yes	
RMSEA	(45)	<0.08	0.011	Yes	
SRMR	(46)	<0.08	0.0394	Yes	
NFI	(47)	>0.80	0.938	Yes	
IFI	(48)	>0.0.90	0.999	Yes	
TLI	(49)	>0.0.90	0.998	Yes	
CFI	(50)	>0.0.90	0.999	Yes	
**Alpha, composite reliability and validity analysis**					
**Construct**	**Items**	**Loading**	**Alpha**	**CR**	**AVE**
		**>0.704**	**>0.7**	**>0.7**	**>0.5**
Sustainable supply chain strategy	SSCS_1	0.797	0.862	0.862	0.676
	SSCS_2	0.851			
	SSCS_3	0.818			
Sustainable supply chain practice	SSCP_1	0.800	0.896	0.897	0.635
	SSCP_2	0.822			
	SSCP_3	0.799			
	SSCP_4	0.797			
	SSCP_5	0.764			
Sustainable competitive advantage	SCA_1	0.832	0.915	0.915	0.683
	SCA_2	0.811			
	SCA_3	0.827			
	SCA_4	0.844			
	SCA_5	0.818			
Sustainability inhibitors	SI_1	0.782	0.893	0.893	0.625
	SI_2	0.793			
	SI_3	0.793			
	SI_4	0.796			
	SI_5	0.789			

**Figure 2:**
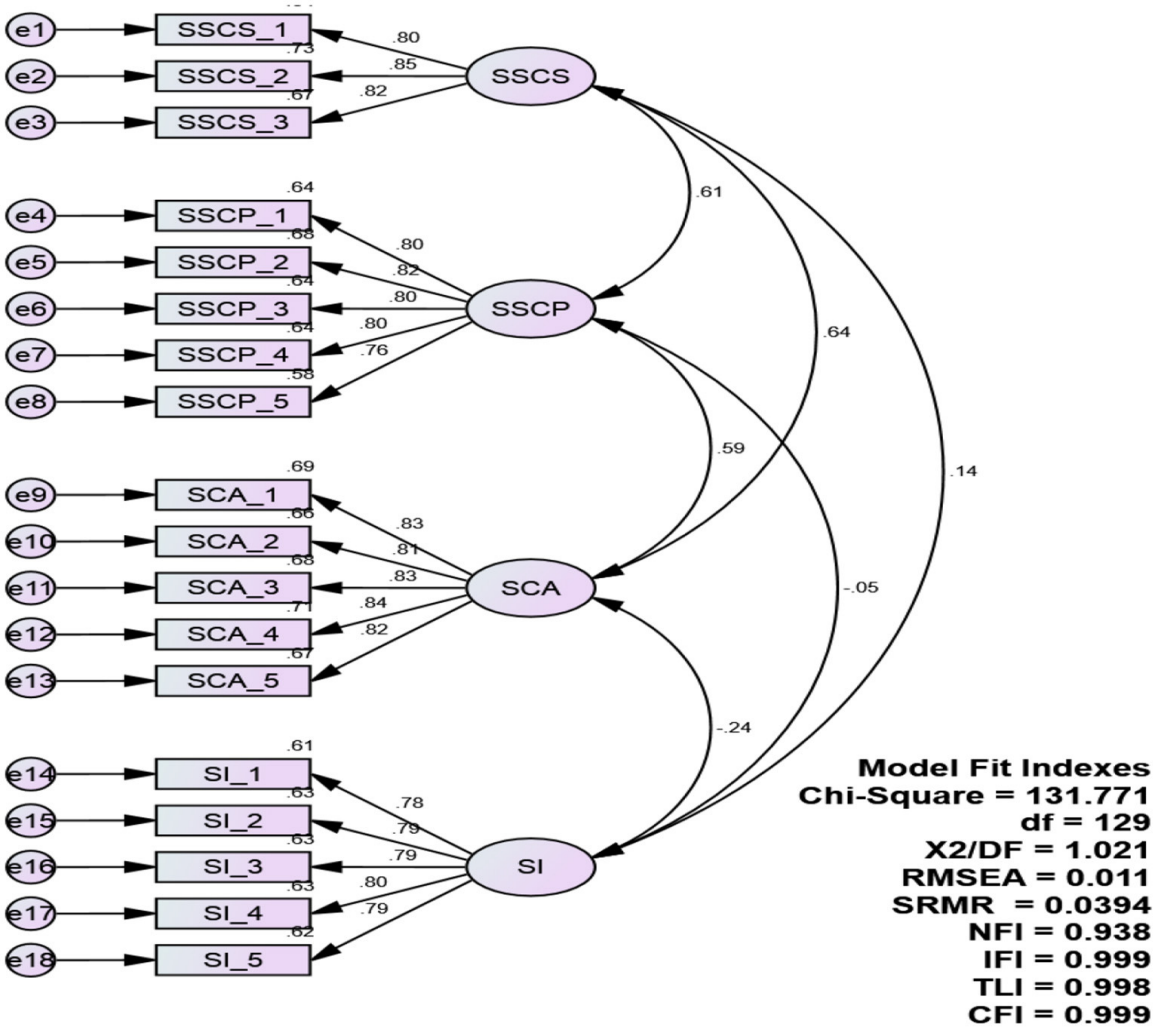
Graphical representation of assessment of measurement model.

Figure 2 depicts the CFA - measurement model with a factor loading of each item above 0.4 (as shown in Table 2), confirming convergent and content validity.

## Results

Results of descriptive statistics (mean and standard deviation) correlation and discriminant validity analysis of the study variables are reported in Table 3. The result depicts values within the range as suggested by Hair (51). As shown sustainable competitive advantage is significantly related to sustainable supply chain strategy (r = 0.641, *p* < 0.05), sustainable supply chain practice (r = 0.594, *p* < 0.05) and sustainability inhibitors (r = −0.239, *p* < 0.05). Similarly, sustainable supply chain strategy is significantly related to sustainable supply chain practice (r = 0.607, *p* < 0.05) and sustainability inhibitors (r = 0.142, *p* < 0.05).

**Table 3:** Discriminant validity analysis (Fornel Larcker and HTMT).

**Constructs**	**Mean**	**SD**	**1**	**2**	**3**	**4**
1. Sustainable competitive advantage	3.44	1.026	(0.826)			
2. Sustainable supply chain strategy	3.38	0.854	0.641**	(0.822)		
3. Sustainable supply chain practice	3.21	0.919	0.594**	0.607**	(0.797)	
4. Sustainability inhibitors	3.29	0.852	−0.239**	0.142**	−0.054**	(0.791)

* *Indicates significant paths:*

** *p <0.001*.

### Hypothesis Testing

Results of hypothesis H1 H2, H3, and H3a (mediation analysis) is shown in Table 4. Hypothesis 1 states that sustainable supply chain strategy is positively related to sustainable competitive advantage with β = 0.484, *p* < 0.001; Hypothesis 2 states that sustainable supply chain strategy is positively related to sustainable supply chain practices with β = 0.693, *p* < 0.001; whereas Hypothesis 3 states that sustainable supply chain practices are positively related to sustainable competitive advantage with β = 0.320, *p* < 0.001. Hence Hypothesis 1, 2, and 3 are statistically proven. For mediation analysis, indirect effects were calculated. Table 4 shows that sustainable supply chain practices mediate the relationship between sustainable supply chain strategy and sustainable competitive advantage with β = 0.222, *p* < 0.001. Hence hypothesis 3a is proved.

**Table 4:** Hypotheses testing direct and indirect effect.

**Hypothesis**	**Direct** **relationships**	**Std.** ***beta***	**Std.** **error**	***T*** **values**	***P*** **values**
H1	SSCS **→** SCA	0.484	0.09	7.126	***
H2	SSCS → SSCP	0.693	0.04	13.846	***
H3	SSCP → SCA	0.32	0.082	4.713	***
H3a	SSCS → SSCP → SCA	0.222	0.067	3.313	***

* *Indicates significant paths:*

*** *p <0.001*.

**Figure 3:**
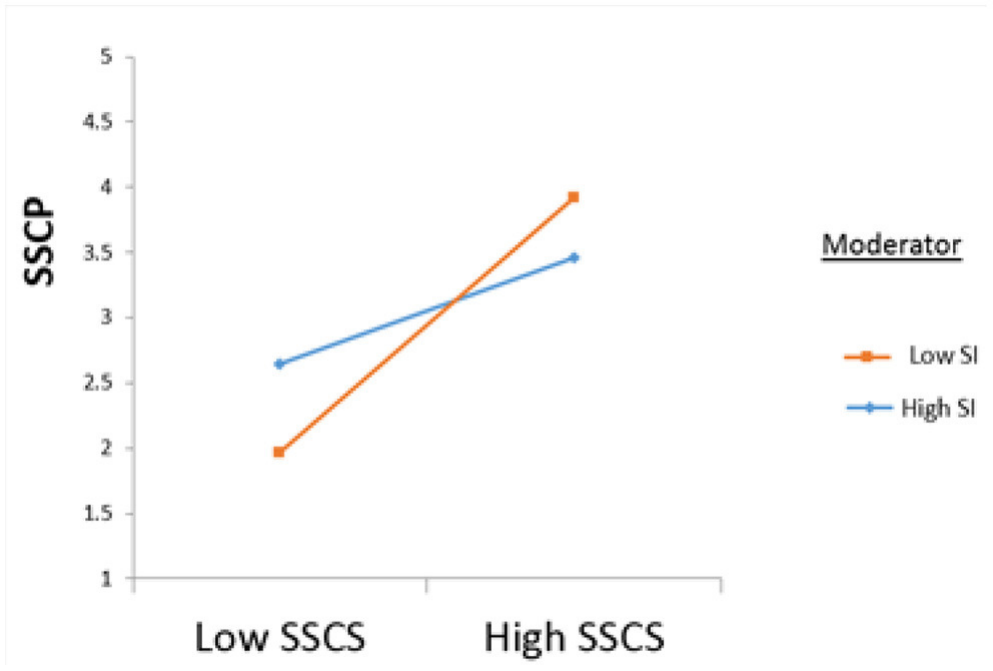
Interaction Effect SSCS*SI → SSCP.

In line with hypothesis 4, the interaction term of sustainable supply chain strategy and sustainability inhibitors was significant (β = 0.315. *p* < 0.05). Moreover, Table 5 illustrates that the relationship between sustainable supply chain strategy and sustainable supply chain practices strengthens when sustainability inhibitors are low (β = 0.976, *p* < 0.001). Figure 3 shows the interaction effect of SSCS^*^SIF0E8SSCP.

**Table 5:** Interaction and conditional effects.

**Hypothesis**	**Interaction effects**	**Std. *Beta***	**Std. Error**	***T* values**	***P* values**
**H4**	Interaction SSCS*SI → SSCP	0.315	0.0634	4.968	***
**Conditional effects**					
	**Level of the moderator**	**Effects**	**Boot SE**	**LLCI**	**ULCI**
	−1 Std Dev	0.976***	0.069	0.839	1.114
	Mean	0.693***	0.050	0.593	0.793
	+1 Std Dev	0.410***	0.070	0.271	0.547

* *Indicates significant paths:*

*** *p <0.001*.

## Discussion

In recent years, sustainability has gained attention in business studies and practices because of rapidly decreasing reserves, concerns about wealth disparities, and firm social and environmental responsibilities (52–54). Intense pressure from various governmental authorities, consumers, suppliers, partners, and local and global communities has encouraged industries to include people, processes, and environmental issues in recent business operations (55). Sustainable supply chain management (SSCM) is a new and rapidly emerging study topic for scholars and practitioners (56). Previous literature reveals that organizations are developing and deploying the best strategies for implementing SSCM practices and policies. In this regard, an effort has been made in the current study to establish a comprehensive framework for assessing the relationship between strategies and performance, which may be valuable to practitioners and managers involved in the SSCM field. In short, this research investigates first: the relationship between sustainable supply chain strategy and sustainable competitive advantage; second: sustainable supply chain practices mediate the relationship between sustainable supply chain strategy and sustainable competitive advantage; third: the moderating role of sustainability inhibitors between sustainable supply chain strategy and sustainable supply chain practices.

Mediation and moderation analyses were conducted using PLS-SEM to check the proposed hypothesis statistically. The results revealed that a sustainable supply chain strategy is positively and significantly associated with sustainable supply chain practices (β = 0.693, *p* < =0.05) and sustainable competitive advantage (β= 0.484, *p* < =0.05); sustainable supply chain practices are positively associated with sustainable competitive advantage (β = 0.320, *p* < =0.05). Henceforth hypotheses H1, H2, and H3 were accepted. Statistically, results revealed that sustainable supply chain practice mediates the relationship between sustainable strategy and competitive advantage; in addition, sustainability inhibitors moderate the relationship between sustainable supply chain strategy and practice. Hence Hypothesis H3a and H4 are approved.

## Conclusion

As a high pollutant with high waste and emission consumption industry, the sustainable strategy will help pharmaceutical supply chain managers improve their overall operations to become smarter, more adaptable, and sustainable in the long run, resulting in better company reputations and sustainable competitive advantages. In the current research, an attempt has been made to analyse the relationship between sustainable supply chain strategy, sustainability inhibitors, sustainable supply chain practices, and sustainable competitive advantage. The findings derived from statistical analysis reveal that sustainable supply chain practice mediates the relationship between sustainable supply chain strategy and sustainable completive advantage. In contrast, sustainability inhibitors moderate the relationship between sustainable strategy and competitive advantage. This study will help managers in developing strategies that will promote sustainable supply chain strategies.

## Data Availability Statement

The raw data supporting the conclusions of this article will be made available by the authors, without undue reservation. The datasets used and/or analyzed during the current study are available from the corresponding author on reasonable request.

## Ethics Statement

Ethical review and approval was not required for the study on human participants in accordance with the local legislation and institutional requirements. The patients/participants provided their written informed consent to participate in this study.

## Author Contributions

All authors listed have made a substantial, direct, and intellectual contribution to the work and approved it for publication.

## Funding

We acknowledge the financial support from the National Natural Science Foundation of China (Grant No: 71974102) and from the Philosophy; Social Science Fund of Tianjin City, China (Grant No: TJYJ20-012).

## Conflict of Interest

The authors declare that the research was conducted in the absence of any commercial or financial relationships that could be construed as a potential conflict of interest.

## Publisher's Note

All claims expressed in this article are solely those of the authors and do not necessarily represent those of their affiliated organizations, or those of the publisher, the editors and the reviewers. Any product that may be evaluated in this article, or claim that may be made by its manufacturer, is not guaranteed or endorsed by the publisher.
